# Oxadiazole-Based Fluorescent Dyes: Photophysical Characterization and Dual Application in Latent Fingerprint Detection and Security Inks

**DOI:** 10.1007/s10895-025-04441-5

**Published:** 2025-07-23

**Authors:** Safa A. Badawy, Abdullah I. Kamel, Ahmed E. Soliman, Mostafa M. Abdul Wahab, Anas M. Khader, Mahmoud E. Abdelgawad, Elaria A. Ibrahim, Basant A. Mostafa, Karim M. Elmezain, Mohamed R. Elmorsy

**Affiliations:** 1 Department of Chemistry, Faculty of Science, Mansoura University, Mansoura, 35516 Egypt; 2 Department of Chemistry, Faculty of Science, New Mansoura University, New Mansoura, 35712 Egypt

**Keywords:** 1,3,4-Oxadiazole dye, Security ink formulation, Thermal stability

## Abstract

In this study, a series of novel oxadiazole-based fluorescent dyes were synthesized and thoroughly characterized through spectroscopic analysis, theoretical modeling, and application testing. The structural design incorporated donor–acceptor motifs to promote intramolecular charge transfer (ICT), resulting in strong fluorescence across the violet-to-green spectral region (363–536 nm) with large Stokes shifts. The photophysical behavior of the dyes was investigated in solvents of varying polarity, revealing distinct solvatochromic effects and enhanced emission efficiency, particularly for diphenylamine-substituted derivatives. Density Functional Theory (DFT) calculations supported experimental observations, confirming frontier orbital distributions consistent with ICT. Thermal stability was confirmed via thermogravimetric analysis, with decomposition temperatures exceeding 250 °C. To explore practical utility, the most emissive dyes were successfully embedded into oxadiazole-based microparticles and tested for latent fingerprint development on porous and non-porous surfaces. Additionally, water-based ink formulations prepared from these dyes demonstrated excellent stability and visibility under UV light, making them suitable for covert writing and anti-counterfeiting applications. The combined structural, optical, and application-oriented results highlight the potential of these oxadiazole derivatives as multifunctional fluorescent materials for forensic and security technologies.

**Supplementary Information:**

The online version contains supplementary material available at 10.1007/s10895-025-04441-5.

## Introduction

**Fig. 1:**
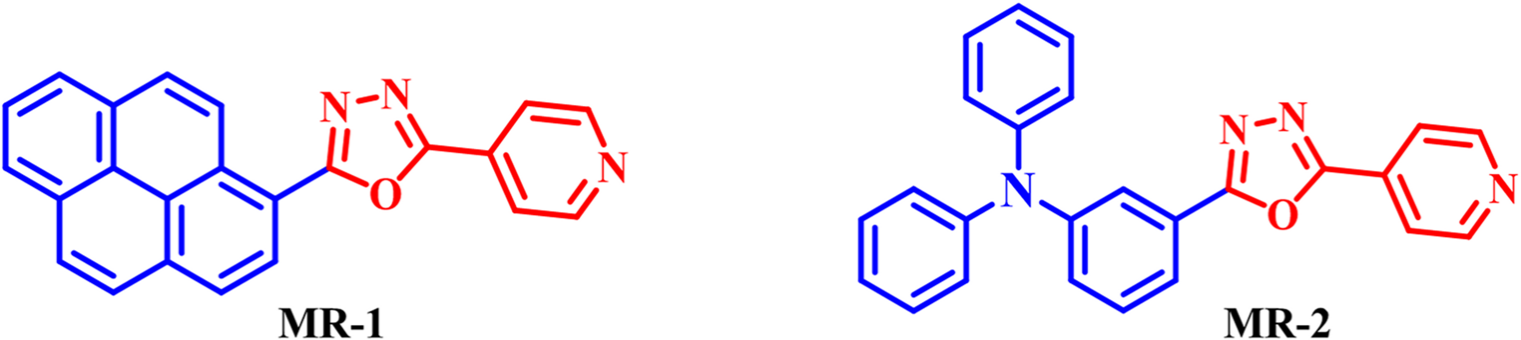
Molecular structure for fluorescent compound **MR-1-2**

Fluorescent organic compounds are widely used in chemical sensing, imaging, and anti-counterfeiting technologies due to their high emission efficiency, structural versatility, and sensitivity to local environments [1–3]. Their ability to emit strong, tunable fluorescence makes them valuable in both solution-phase and solid-state applications, particularly when molecular design allows for tailored electronic interactions. Among fluorescent heterocycles, 1,3,4-oxadiazole derivatives have received particular attention. These nitrogen–oxygen-containing five-membered rings function as strong electron-accepting units and are commonly used to build donor–acceptor systems [4–6]. Oxadiazoles exhibit high thermal and photochemical stability, excellent electron mobility, and significant electron affinity, making them effective as electron-transport and injection layers in organic light-emitting devices (OLEDs) [7, 8]. When paired with electron-rich donor units, such systems often show enhanced intramolecular charge transfer (ICT), leading to solvent-sensitive emission and large Stokes shifts [9]. Additionally, some oxadiazole-based systems are capable of excited-state intramolecular proton transfer (ESIPT), a process that produces dual emission bands and suppresses self-quenching, thereby increasing quantum efficiency [10, 11]. The combination of ICT and ESIPT mechanisms often results in broad, red-shifted fluorescence with minimal overlap between absorption and emission, ideal for imaging and sensing applications [12]. Beyond their well-established role in optoelectronics, oxadiazole derivatives have also shown great promise as fluorescent components in security inks. Their high luminescence efficiency, strong environmental sensitivity, and robust photostability make them suitable for inks that remain invisible under ambient light but emit brightly under UV illumination [13–15]. Such inks are of growing interest in anti-counterfeiting, covert labeling, and encrypted information storage, where reliability and tunability are critical. Oxadiazole-based dyes can be incorporated into aqueous or polymer-based systems, and their emission can be modulated by external factors such as polarity or pH, enabling the development of stimuli-responsive security inks [16]. When embedded into biopolymer carriers such as starch, these dyes also benefit from improved environmental compatibility, ease of application, and increased stability [17]. To extend their practical use, particularly in portable or field-deployable formats, embedding fluorescent dyes into polymeric carriers has become a promising strategy. Organic compounds, in particular, offers a low-cost, biodegradable, and non-toxic platform for dye encapsulation, enhancing both stability and surface adhesion. This approach has been especially useful in forensic science, where fluorescent microparticles allow for high-contrast visualization of latent fingerprints on various porous and non-porous surfaces [17]. In this work, we report the synthesis and photophysical characterization of oxadiazole-based fluorescent dyes **MR-1-2** as shown in Fig. 1 engineered for strong and tunable emission. Their optical behavior was investigated in solvents of varying polarity and interpreted using density functional theory (DFT) calculations. The most emissive derivatives were successfully embedded into oxadiazole-based microparticles and tested in two practical applications: (i) latent fingerprint visualization under UV light, and (ii) fluorescent security ink formulation for covert data encoding. These results demonstrate the dual utility of oxadiazole dyes in security and forensic technologies and offer a strategy for developing multifunctional, environmentally friendly fluorescent materials.

## Experimental

### Synthesis

#### *Synthesis of Pyrene Hydrazide (3)*

An equimolar ethanolic solution of acid hydrazide (0.1 mol) **(1)** and pyrene aldehyde (0.1 mol) (2) was prepared, and a few drops of glacial acetic acid were added to the mixture. The solution was then refluxed for 6 h, allowed to cool, and subsequently filtered. The crude product was recrystallized from methanol, resulting in the formation of pyrene carbohydrazide [18]. 

#### *Synthesis of 2-(pyren-1-yl)-5-(pyridin-4-yl)-1, 3, 4-oxadiazole (MR-1)*

A solution of pyrene carbohydrazides (**3**) (5.00 g) and potassium permanganate (5.00 g) in acetone (50 mL) was stirred at 50 °C for 4 h. Once the reaction was complete, the mixture was concentrated under reduced pressure to remove acetone. The residue was then added to a saturated aqueous solution of sodium sulfide (30 mL) and extracted with dichloromethane (30 mL) and ethyl acetate (150 mL). The combined organic extracts were dried over magnesium sulfate, filtered, and concentrated under reduced pressure. The crude product was then chromatographed using a mixture of ethyl acetate and hexane (1:1) on silica gel. Finally, the organic solvents were evaporated to yield pyrene-oxadiazoles (**MR-1**).

Yellow crystal, yield: 67%; m.p: 192 °C; IR (KBr): *ν*_*max*_ 1589 (C = N) cm^− 1^. ^1^HNMR (DMSO-*d*_6_, 400 MHz): *δ* 8.30 (d, *J* = 4.00 Hz, 2 H, Ar-H), 8.38–8.40 (m, 2 H, Ar-H), 8.45–8.47 (m, 2 H, Ar-H), 8.50 (q, *J* = 4.00 Hz, 2 H, Ar-H), 8.85 (d, *J* = 4.00 Hz, 1H, Ar-H), 8.90 (d, *J* = 4.00 Hz, 2 H, Ar-H), 8.99 ppm (d, *J* = 8.00 Hz, 2 H, Ar-H).^13^CNMR(DMSO-*d*_6_): 123.4, 123.5, 124.8, 125.4, 126.4, 127.2 (4 C), 127.6, 128.6, 129.0, 129.1, 130.0, 130.9, 131.7 (2 C), 133.0, 133.3, 150.3 (2 C), 163.3, 165.4 ppm. Analysis calcd. For C_23_H1_3_N_3_O (347.38): C, 79.53; H, 3.77; N, 12.10%. Found: C, 79.43; H, 3.72; N, 12.14%.

#### *Synthesis of Triphenyl Amine Hydrazide (5)*

A few drops of glacial acetic acid were added to an equimolar ethanolic solution containing triphenylamine aldehyde **(4)** (0.1 mol) and acid hydrazide (0.1 mol) **(2).** After that, the mixture was refluxed for six hours. After that, it was cooled and filtered. Pyrene carbohydrazides were obtained by recrystallizing the crude product from methanol **(5).** [19]

#### *Synthesis of N, N-diphenyl-3-(5-(pyridin-4-yl)-1,3,4-oxadiazol-2-yl)aniline (MR-2)*

A combination of 5.00 g of potassium permanganate and 5.00 g of triphenylamine carbohydrazides was dissolved in 50 mL of acetone and stirred for four hours at a temperature of 50 °C. Once the reaction was complete, the acetone was eliminated from the mixture by evaporating it under reduced pressure. Subsequently, a saturated sodium sulfide solution (30 mL) was introduced to the remaining residue. The resulting mixture was then extracted with 30 mL of dichloromethane and 150 mL of ethyl acetate. After drying the organic extracts over magnesium sulfate, they were filtered and concentrated under reduced pressure. The crude product was then purified by chromatography on silica gel using a solvent blend of ethyl acetate and hexane in a 1:1 ratio. The final compound, triphenylamine-oxadiazoles (**MR-2**), was obtained by removing the organic solvents through evaporation.

Yellow crystal, yield: 78%; m.p: 192 °C; IR (KBr): *ν*_*max*_ 1589 (C = N) cm^− 1^. ^1^HNMR (DMSO-*d*_6_, 400 MHz): *δ* 6.88–6.89 (m, 1H, Ar-H), 7.03 (d, *J* = 8.00 Hz, 2 H, Ar-H), 7.18–7.19 (m, 6 H, Ar-H), 7.41 (d, *J* = 8.00 Hz, 3 H, Ar-H), 7.79 (d, *J* = 8.00 Hz, 2 H, Ar-H), 7.90 (d, *J* = 8.00 Hz, 2 H, Ar-H) 8.86 ppm (d, *J* = 4.00 Hz, 2 H, NCH_2_).^13^CNMR(DMSO-*d*_6_): *δ* 122.8, 123.4 (2 C), 124.0, 124.8, 126.2 (4 C), 128.3 (2 C), 129.8 (4 C), 129.9, 130.2, 133.3, 143.3, 145.4 (2 C), 150.3 (2 C), 165.4, 165.8 ppm. Analysis calcd. For C_25_H_18_N_4_O (390.15): C, 76.91; H, 4.65; N, 14.35%. Found: C, 76.97; H, 4.57; N, 14.30%.

## Results and Discussion

**Scheme 1:**
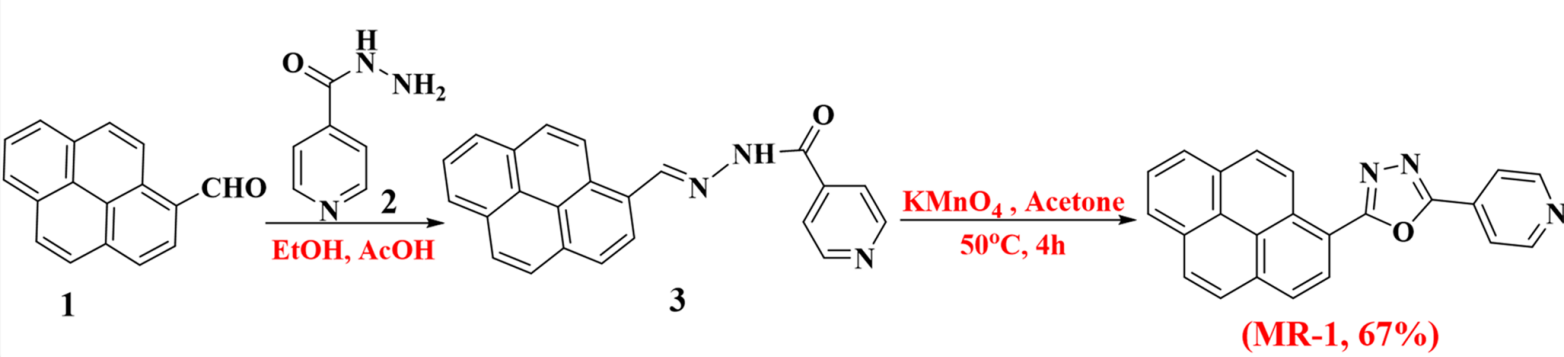
Synthesis of MR-1 compound

Pyrene-1-carbaldehyde (1) and 4-(diphenylamine) benzaldehyde (4) were refluxed with acid hydrazides (2) in ethanol, leading to the formation of Pyrene carbohydrazide and triphenylamine carbohydrazide, referred to as compounds **3** and **5**. Subsequently, the Pyrene carbohydrazides were reacted with aromatic aldehydes in the presence of potassium permanganate in acetone, resulting in the production of pyrene-oxadiazole (**MR-1**), as illustrated in Scheme 1. The structures of the synthesized compounds were characterized using IR and ^1^H NMR spectroscopy techniques. In the IR spectra, the stretching vibrations of the C = N bond were observed at 1589 cm^− 1^. The ^1^H NMR spectra of **MR-1** displayed a doublet signal at *δ* 8.30 ppm, corresponding to the aromatic protons, and a quartet signal at *δ* 8.50 ppm, also assigned to aromatic protons. Additionally, a doublet signal at *δ* 8.90 ppm, characteristic of the azomethine (N = CH) protons, was noted. The ^13^C NMR signals for the carbon attached to the pyridine ring of **MR-1** were observed at *δ* 150.3 ppm, while the signals for the C = N bonds were found at *δ* 163.3 and 165.4 ppm.

**Scheme 2:**
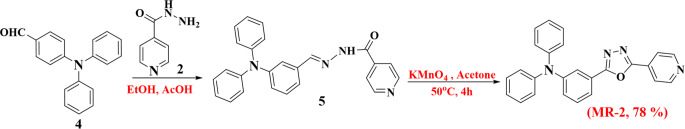
Synthesis of MR-2 compound

The triphenylamine carbohydrazides reacted with aromatic aldehydes in the presence of potassium permanganate and acetone to produce triphenylamine-Oxadiazoles (**MR-2**), as shown in Scheme 2. The infrared (IR) spectrum of **MR-2** displays a peak at 1589 cm^− 1^, indicating the presence of a C = N moiety. In the ^1^H NMR spectrum, the protons linked to the pyridine ring appear as a doublet at *δ* 8.86 ppm. Furthermore, six aromatic protons can be seen between *δ* 7.18 and 7.19 ppm. The ^13^C NMR signals for the carbon attached to the pyridine ring of **MR-2** are observed at *δ* 150.3 ppm, while the signals for C = N are recorded at *δ* 165.8 ppm.

### Spectroscopic Properties for Compound MR-1-2

**Fig. 2:**
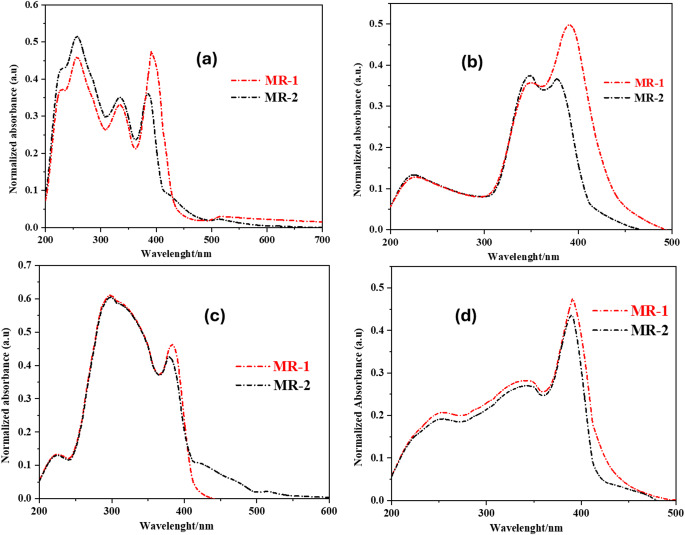
Normalized absorption spectra of MR-1-2 in, (**a**) Acetone, (**b**) DMSO, (**c**) Methanol, (**d**) Dichloromethane

The absorption spectra of the new 1,3,4-oxadiazole derivatives were recorded in various solvents (Fig. 2) and the relevant data are summarized in Table 1.

**Table 1:** Relevant data of absorption spectra for MR-1-2

Derivative	Solvent	Absorption UV–vis λ_max_^a^ (nm)
MR-1	Acetone	391
	DMSO	389
	Methanol	390
	Dichloromethane	384
MR-2	Acetone	386
	DMSO	376
	Methanol	387
	Dichloromethane	376

^a^ Absorption maximum band corresponding to π → π* transition

The solvent-dependent UV–Vis absorption spectra of **MR-1** and **MR-2** were recorded in acetone, DMSO, methanol, and dichloromethane to investigate their electronic transitions and the influence of solvent polarity on their photophysical properties. The normalized absorption spectra are shown in Fig. 1(a–d). In **acetone** (Fig. 1a), both **MR-1** and **MR-2** exhibit well-defined absorption bands in the UV-visible region. **MR-1** shows multiple peaks, with the most intense band centered near **391 nm**, suggesting π→ π* transition associated with the conjugated chromophore. **MR-2** displays a similar profile but with its maximum slightly blue-shifted to **386 nm** and of higher intensity, reflecting stronger absorption and possibly enhanced electron delocalization [20]. Both compounds also show higher-energy bands below 300 nm, corresponding to transitions from deeper-lying orbitals. In **DMSO** (Fig. 1b), MR-1 maintains a strong, sharp absorption with a λ_max_ at 389 nm, while **MR-2** exhibits a broader and weaker absorption band, centered around 376 nm. The more significant blue shift in **MR-2** suggests a greater sensitivity to solvent polarity and a reduced stabilization of the excited state in this highly polar aprotic environment. This observation supports the hypothesis that **MR-2** undergoes stronger solvent-dependent interactions due to its more pronounced intramolecular charge transfer (ICT) nature [21]. In methanol (Fig. 1c), both compounds show red-shifted absorption bands compared to DMSO, with **MR-1** absorbing at 390 nm and **MR-2** at 388 nm. The proximity of these peaks implies that protic solvents like methanol stabilize the excited states more effectively, particularly for **MR-2**, narrowing the energy gap. The absorption profile of **MR-2** is broader and slightly more intense in methanol, which may facilitate stronger fluorescence emission in this medium. In **dichloromethane (DCM)** (Fig. 1d), **MR-1** absorbs at 384 nm, and **MR-2** at 379 nm. These shifts indicate a consistent trend: both compounds exhibit blue-shifted absorption in lower-polarity solvents, with **MR-2** always absorbing at slightly shorter wavelengths than **MR-1**. This suggests that the excited state of **MR-2** is more sensitive to the surrounding medium, reflecting its greater ICT contribution [21]. Across all solvents, **MR-2** consistently shows broader and more variable absorption, indicating that it is more responsive to changes in solvent polarity and hydrogen-bonding interactions. This behavior aligns well with its enhanced fluorescence characteristics observed under UV light, as stronger ICT and environmental sensitivity often correlate with increased emission efficiency. Meanwhile, **MR-1** maintains narrower, more consistent peaks, indicating relatively stable optical behavior and lower polarity-dependent variation. These results confirm that both compounds exhibit solvent-dependent optical properties, with **MR-1-2** showing stronger solvatochromism and better suitability for applications where environmental responsiveness and high fluorescence output are desirable, such as in sensing, security printing, or imaging technologies.

**Fig. 3:**
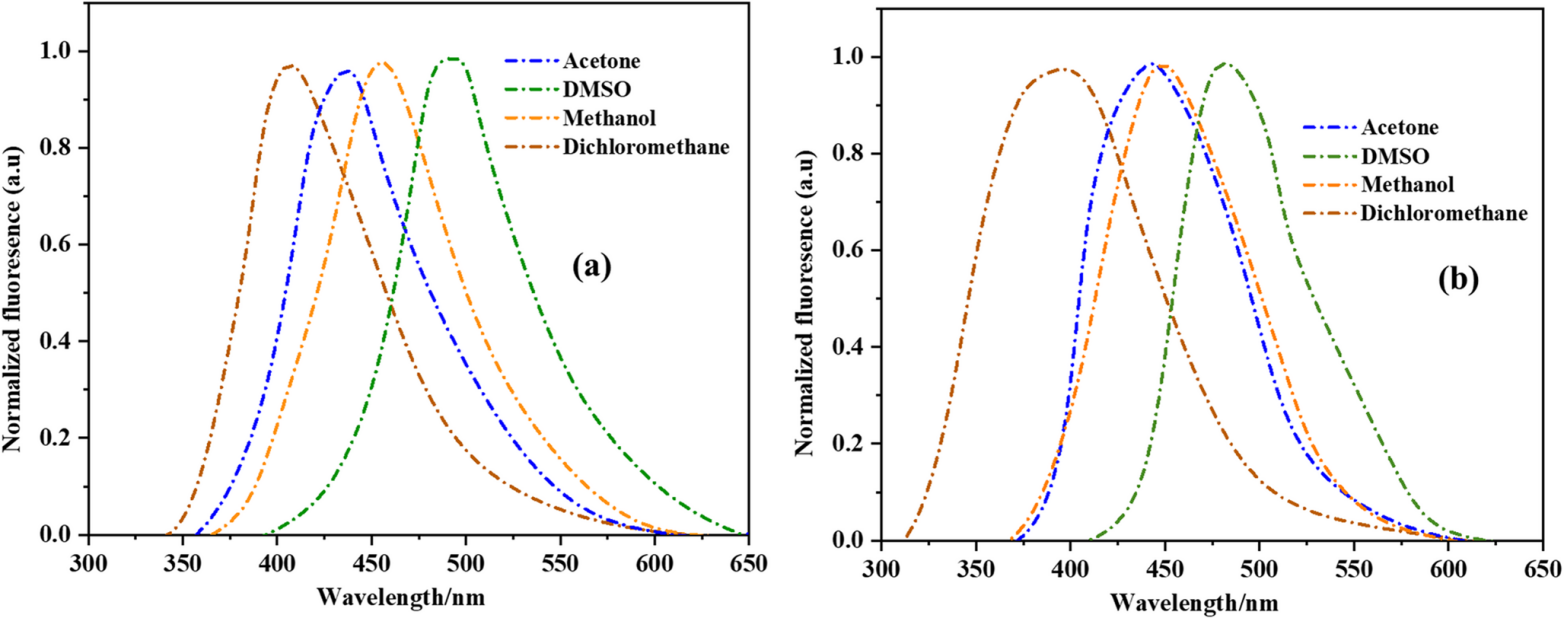
Normalized fluorescence emission spectra of (**a**) **MR-1** and (**b**) **MR-2** in solvents with different polarities

The fluorescence emission spectra of all derivatives were performed in solvents of different polarities and are shown in Fig. 3. The wavelength of maximum absorption of each derivative was used as excitation to obtain the fluorescence spectra. The relevant data are summarized in.

**Table 2:** Relevant data of fluorescence emission spectra for **MR-1-2**

Derivative	Solvent	λ_em_(nm)	ΔλST (nm)
MR-1	Acetone	435	44
	DMSO	492	103
	Methanol	455	65
	Dichloromethane	410	26
MR-2	Acetone	437	51
	DMSO	480	104
	Methanol	450	63
	Dichloromethane	400	24

The fluorescence emission spectra of **MR-1** and **MR-2** were recorded in four solvents acetone, DMSO, methanol, and dichloromethane to assess their photophysical behavior and environmental sensitivity (Fig. 3a and b). The corresponding emission maxima (λem) and Stokes shifts (ΔλST) are summarized in Table 2. **MR-1** contains a pyrene donor linked to a 1,3,4-oxadiazole–pyridine acceptor, forming a modest donor–acceptor (D-A) system [22]. Its emission maxima shift from 410 nm in dichloromethane to 492 nm in DMSO, with Stokes shifts increasing from 26 nm to 103 nm as solvent polarity increases. These bathochromic shifts reflect stabilization of the excited state by polar solvents, indicative of moderate intramolecular charge transfer (ICT). The relatively narrow shifts in less polar solvents suggest a more rigid excited-state geometry. **MR-2**, incorporating a stronger triphenylamine donor in the same D–A framework, shows even greater solvent-dependent variation. Its emission maxima range from 400 nm to 480 nm, with Stokes shifts between 24 nm (DCM) and 104 nm (DMSO). The larger shifts and broader emission bands indicate enhanced ICT behavior and greater excited-state relaxation, driven by its more polarized structure [23]. Importantly, both **MR-1** and **MR-2** exhibit strong fluorescence in all solvents tested, confirming their suitability as effective emissive materials. **MR-2**, in particular, demonstrates higher sensitivity to the surrounding medium and more pronounced fluorescence shifts, consistent with its extended conjugation and stronger donor–acceptor interaction. The high emission intensities observed in both compounds underscore their **good fluorescence ability**, making them promising candidates for applications in sensing, imaging, and security technologies. The structural features of **MR-1-2** especially the stronger donor in **MR-2** directly influence their fluorescence response. Both compounds display efficient light emission, but **MR-2** stands out for its greater solvatochromic behavior and superior fluorescence tunability, driven by its stronger intramolecular charge transfer character [24]. Both compounds demonstrate good fluorescence ability in all tested solvents, with **MR-2** showing superior performance in terms of emission tunability and environmental responsiveness. These properties make **MR-2** particularly promising for applications in fluorescent sensing, polarity mapping, and optical security materials, while **MR-1** offers stability in environments requiring consistent optical response.

### Theoretical Calculations

#### Electronic Density of Frontier Orbitals for Compounds MR-1-2

**Fig. 4:**
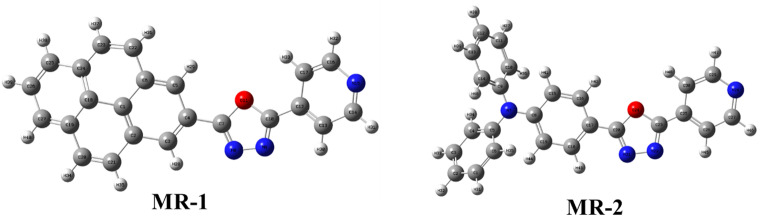
Optimized structure for compounds **MR-1-2**

**Fig. 5:**
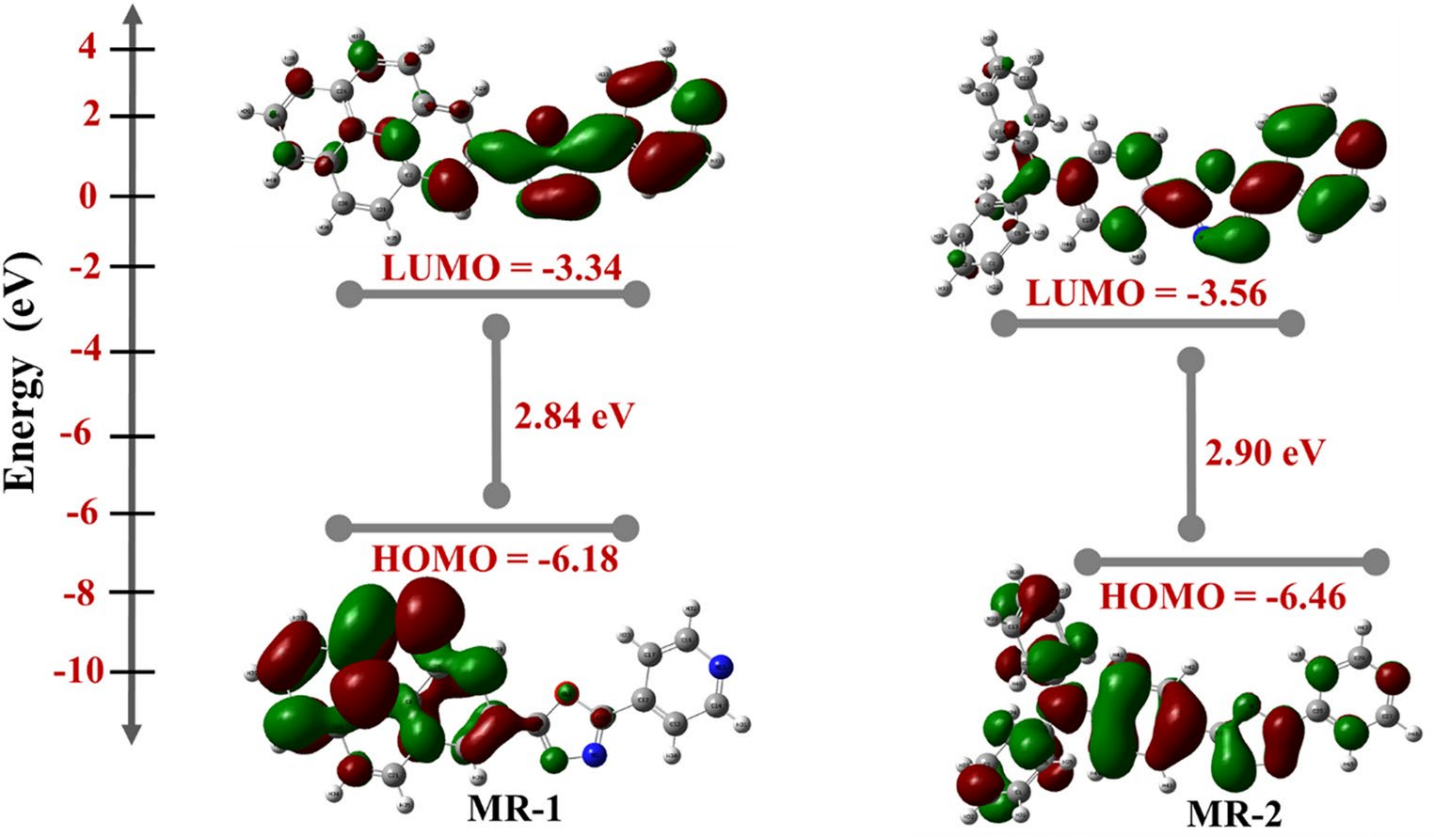
Electron density and energy levels of FMO for **MR-1-2** compounds

Theoretical calculations were performed with Density Functional Theory (DFT) using Gaussian 09 software package [25] in order to better understand the photophysical properties of the synthesized benzazole derivatives. The geometry structures of all derivatives were optimized at the B3LYP/6-31G (d) level [26, 27]. To understand the photophysical behavior of the synthesized dyes **MR-1** and **MR-2**, particularly their fluorescence activity, we analyzed their frontier molecular orbitals using DFT calculations. The optimized structures and HOMO-LUMO energy levels and electronic density distributions are presented in Figs. 4 and 5. The FMO spatial distributions further support the donor–π–acceptor (**D-π-A**) architecture of both dyes. In **MR-1**, the HOMO is primarily localized over the electron-rich pyrene moiety, while the LUMO is concentrated on the oxadiazole-pyridine acceptor segment.

Similarly, in **MR-2**, the HOMO is localized on the triphenylamine donor, and the LUMO extends over the oxadiazole-pyridine unit. This clear separation of frontier orbitals enhances intramolecular charge transfer upon excitation, which is a hallmark of fluorescent molecules with efficient emission characteristics. the presence of the 1,3,4-oxadiazole moiety in both compounds contributes significantly to their electron-accepting ability and photostability, which are key for high fluorescence efficiency. The more pronounced ICT in **MR-2**, as reflected by its lower energy gap and broader orbital delocalization, suggests a stronger fluorescence response compared to **MR-1**. This makes **MR-1 and MR-2** particularly promising for applications in fluorescent sensors or organic light-emitting devices (OLEDs) [28]. The electronic structure analysis demonstrates that both **MR-1** and **MR-2** possess favorable orbital alignments and bandgaps for fluorescence. The more efficient charge separation and lower energy gap of **MR-2** suggest that it may exhibit stronger fluorescence intensity and longer emission wavelength than **MR-**1, in line with typical D-π-A fluorophore behavior.

#### Quantum Chemical Parameter for Oxadiazole Compounds MR-1-2

To investigate the molecular structures of the oxadiazole **MR-1-2**. sensitizers, we employed density functional theory (TD-DFT) calculations using the B3LYP/6-31G (d) function and basis set. These computations were carried out using the Gaussian 09 software package. Theoretical calculations were all performed in water and gas phase [29, 30]. Table 3 displays the relevant data, and the calculations were conducted in accordance with Eqs. (**1–5**).

1 $${IP = - {E_{HOMO}}}$$

2 $$EA = - {E_{LUMO}}$$

3 $$\eta \, = \,\left( {{{{E_{LUMO}} - {E_{HOMO}}} \over 2}} \right)$$

4 $$s\, = \,\left( {1/{\rm{ }}\eta } \right)$$

5 $$\chi \, = \, - \,\mu \, = \, - \,\left( {{E_{LUMO}}\, + \,{E_{HOMO}}} \right)/2$$

The quantum chemical descriptors derived from DFT calculations offer valuable insights into the electronic structure, reactivity, and fluorescence potential of the **MR-1** and **MR-2** compounds. These parameters include the energies of the frontier molecular orbitals (HOMO and LUMO), ionization potential (IP), electron affinity (EA), energy gap (E_0 − 0_), chemical hardness (η), softness (S), chemical potential (µ), and electronegativity (χ), all of which are summarized in Table 2. The HOMO and LUMO energies for **MR-1** are − 6.18 eV and − 3.34 eV, respectively, while those for **MR-2** are − 6.46 eV and − 3.56 eV. These values result in (*E*_*0 − 0*_) of 2.84 eV for **MR-1** and 2.90 eV for **MR-2.** Although **MR-2** has a slightly wider bandgap, its deeper HOMO and more stabilized LUMO suggest a greater potential for charge separation and stabilization of the excited state. The experimentally determined band gaps from UV–Vis absorption spectra show good agreement with the TD-DFT calculated values. For **MR-1**, the experimental band gap was 2.45 eV, while the theoretical value was 2.61 eV. Similarly, **MR-2** exhibited an experimental gap of 2.51 eV compared to a calculated value of 2.65 eV. The slight differences are within expected ranges and are likely due to solvent effects and the limitations of the computational method. This correlation confirms the validity of the theoretical approach in predicting the electronic properties of the dyes. The higher **ionization potential (IP = 6.46 eV)** and **electron affinity (EA = 3.56 eV)** of MR-2 compared to **MR-1** (IP = 6.18 eV, EA = 2.72 eV) imply that MR-2 is both a stronger electron donor and acceptor. This dual behavior facilitates efficient intramolecular charge transfer (ICT), which is a critical feature for achieving strong and tunable fluorescence emission. In addition, MR-2 exhibits greater **(η = 1.45 eV)** and a more negative **(µ = − 5.01 eV)** than MR-1 (η = 1.42 eV, µ = − 4.45 eV), indicating enhanced electronic stability and lower reactivity toward nonradiative decay pathways. The **electronegativity** of **MR-2** (χ = 5.01 eV) is also higher than that of MR-1 (χ = 4.45 eV), reflecting a stronger tendency to attract electrons, which supports the D–π–A architecture needed for efficient ICT processes. From a photophysical standpoint, compounds with strong ICT character, stabilized excited states, and minimized nonradiative losses generally exhibit higher fluorescence quantum yields and longer emission wavelengths. Although **MR-1** demonstrates a slightly narrower bandgap and higher softness (S = 0.70 eV) than MR-2 (S = 0.68 eV), which may facilitate easier excitation, these advantages are outweighed by the superior excited-state stabilization and ICT efficiency of MR-2. The presence of a more electron-rich donor unit and a stronger electron-accepting moiety in MR-2 enhances this intramolecular charge transfer upon excitation, which is expected to produce a more intense fluorescence response. While both **MR-1** and **MR-2** exhibit promising electronic characteristics for fluorescent dye applications, the overall quantum chemical profile of **MR-**2 marked by its deeper frontier orbitals, higher electron affinity, stronger electronegativity, and more negative chemical potential, suggests that it will exhibit superior fluorescence performance. These features make **MR-2** a more suitable candidate for optoelectronic applications, including devices (OLEDs), bioimaging, and fluorescent sensors.

**Table 3:** Quantum chemical parameters of compound **MR-1-2**

Theoretical	MR-1	MR-2
*HOMO (eV)*	-6.18	-6.46
*LUMO (eV)*	-3.34	-3.56
*E*_*0 − 0*_ *(eV)*	2.84	2.90
*IP (eV)*	6.18	6.46
*EA (eV)*	3.34	3.56
*η (eV)*	1.42	1.45
*S (eV)*	0.70	0.68
*µ (eV)*	-4.45	-5.01
*χ (eV)*	4.45	5.01

Molecular Electrostatic Potential (MEP) Mapping of compound MR-1-2.

**Fig. 6:**
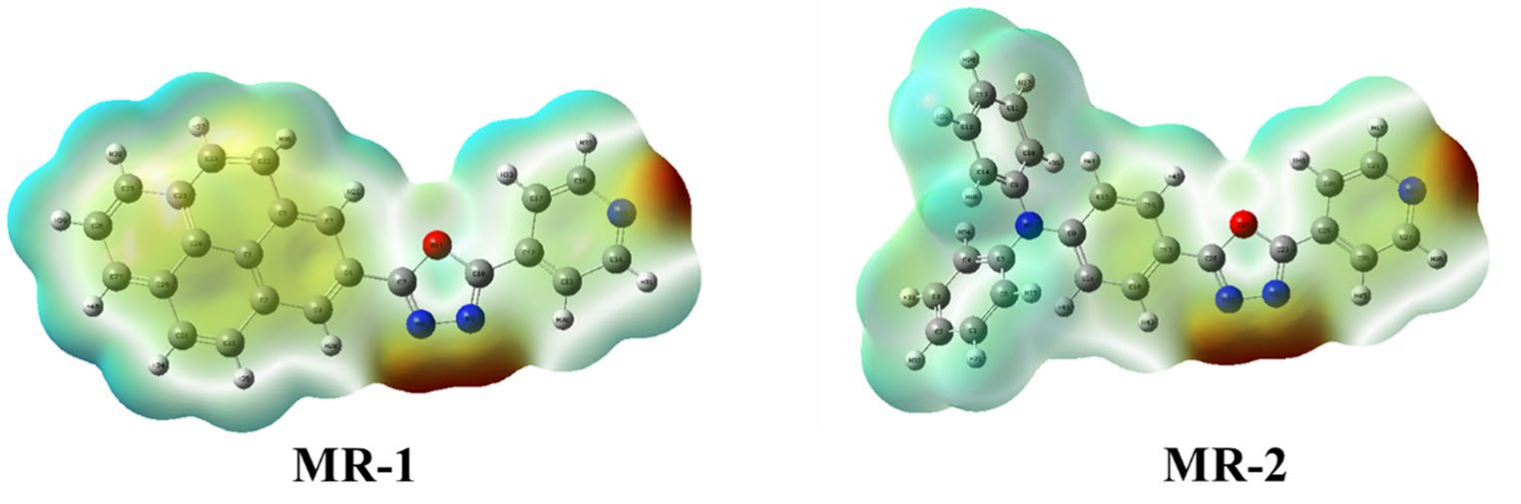
Surface map of electrostatic potential distribution of **MR-1-2**

Electrostatic potential (MEP) mapping is a valuable computational tool to visualize the charge distribution over a molecule’s surface and identify regions susceptible to electrophilic or nucleophilic attack as shown in Fig. 6 [31, 32]. The MEP maps of the **MR-1** and **MR-2** molecules are depicted in Fig. 6. The color-coded surfaces represent different potential values: red regions indicate areas of high electron density (electron-rich), which are favorable for electrophilic attack, while blue to white regions indicate electron-deficient zones, typically involved in nucleophilic interactions or hydrogen bonding. As shown in the MEP maps, both **MR-1** and **MR-2** exhibit pronounced red zones around electronegative atoms such as nitrogen and oxygen, particularly in the oxadiazole and pyridine rings. These regions, rich in electron density, are potential sites for electrophilic interaction. The red coloration around these heteroatoms confirms their electron-withdrawing nature and their role in stabilizing negative charge through resonance and inductive effects. In contrast, the hydrogen-rich areas, especially those bonded to aromatic systems, are shown in light blue to white, indicating regions of positive potential and lower electron density, making them more prone to nucleophilic attack. Notably, **MR-2** shows a more delocalized electrostatic potential distribution across its structure compared to **MR-1**. This broader charge separation reflects its stronger (ICT) character, in agreement with its lower LUMO energy and higher electron affinity discussed previously. The more extended electron-rich regions in **MR-2** further support its enhanced acceptor behavior, contributing to better stabilization of the excited state, which is beneficial for its fluorescence performance [33]. The red regions in the MEP maps highlight areas of high electron density (notably around nitrogen and oxygen atoms in the oxadiazole and pyridine rings), which are potential sites for hydrogen bonding or electrostatic interactions with surface residues (e.g., amino acids, fatty acids) commonly found in fingerprints. Conversely, the blue/white regions indicate electron-deficient areas that could interact with nucleophilic surface sites. These charge distribution patterns suggest that both **MR-1** and **MR-2** have strong potential for non-covalent dye–surface interactions, facilitating effective adherence to both porous and non-porous substrates. Additionally, the broader charge delocalization observed in **MR-2** (due to stronger ICT) may enhance its interaction range and improve fluorescence contrast on complex surfaces.

### Thermogravimetric Analysis for MR-1-2

**Fig. 7:**
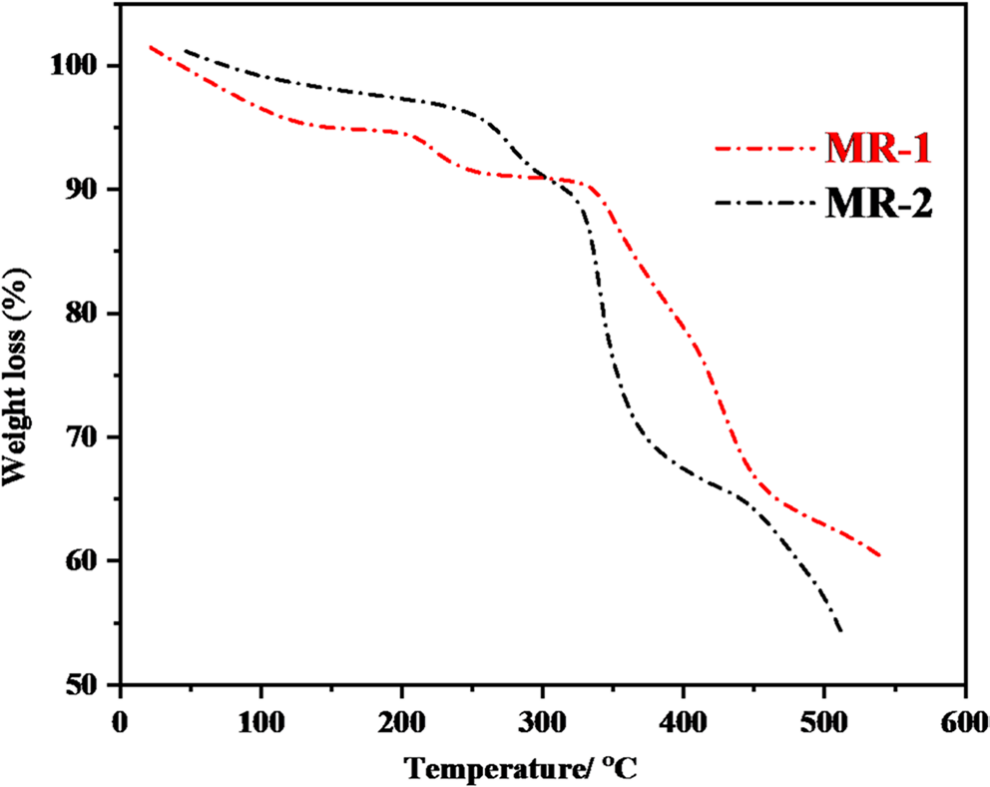
Thermogravimetric of synthesized oxadiazole **MR-1-2**

Figure 7 shows the thermogravimetric analysis (TGA) curves of the synthesized fluorescent dyes **MR-1** and **MR-2**, which provide insight into their thermal stability and decomposition behavior [34]. As shown in Fig. 7, **MR-1**, an initial weight loss is observed between 25 °C and 120 °C, corresponding to the loss of physically adsorbed moisture or volatile components. The presence of polar functional groups such as the oxadiazole and pyridine rings may contribute to the retention of moisture. Following this, **MR-1** exhibits a gradual mass loss from 120 °C to 380 °C, suggesting the onset of molecular fragmentation and the beginning of structural decomposition. A more significant weight loss stage is observed from 380 °C to 500 °C, indicating the complete breakdown of the organic framework and thermal degradation of the dye structure. At 500 °C, approximately 60% of the initial mass remains, indicating a moderate char yield and partial carbonization. In comparison, the TGA curve of **MR-2** shows a similar initial weight loss below 100 °C, attributed to moisture release, followed by a more rapid decomposition phase starting at around 180 °C. The main decomposition stage occurs between 180 °C and 450 °C, which may involve the breakdown of the diphenylamine unit and the oxadiazole–pyridine conjugation system. **MR-2** shows a sharper and earlier onset of degradation compared to **MR-1**, likely due to the presence of more thermally labile bonds in its extended π-conjugated system. At 500 °C, **MR-2** retains slightly less mass than MR-1, with a final residual weight just above **55%**, indicating a relatively lower char yield. Overall, both compounds demonstrate good thermal stability up to 200 °C, making them suitable for practical applications where moderate thermal resistance is required. **MR-1** appears to be marginally more thermally stable than **MR-2**, as reflected by its delayed onset of major decomposition and higher char residue. These findings are consistent with their molecular structures, where MR-1’s pyrene unit offers slightly higher thermal rigidity compared to the more flexible diphenylamine group in **MR-2.** Despite this, both compounds maintain sufficient stability for use in optoelectronic and fluorescence-based applications that operate under ambient or slightly elevated temperatures. The thermal stability of **MR-1** and **MR-2** was evaluated by (TGA), revealing decomposition onset temperatures above 180 °C and char yields exceeding 55% at 500 °C. These values compare favorably with related dyes reported for security and forensic applications, such as ESIPT-based and benzazole derivatives, which typically decompose in the 150–220 °C range [34]. The enhanced thermal resistance of **MR-1** and **MR-2** is attributed to their rigid aromatic frameworks and the presence of electron-withdrawing oxadiazole and pyridine units, making them suitable candidates for use in thermally demanding conditions, including latent fingerprint development and anti-counterfeiting inks.

### Latent Fingerprint Visualization on Different Surfaces using Oxadiazole Compound MR-1

**Fig. 8:**
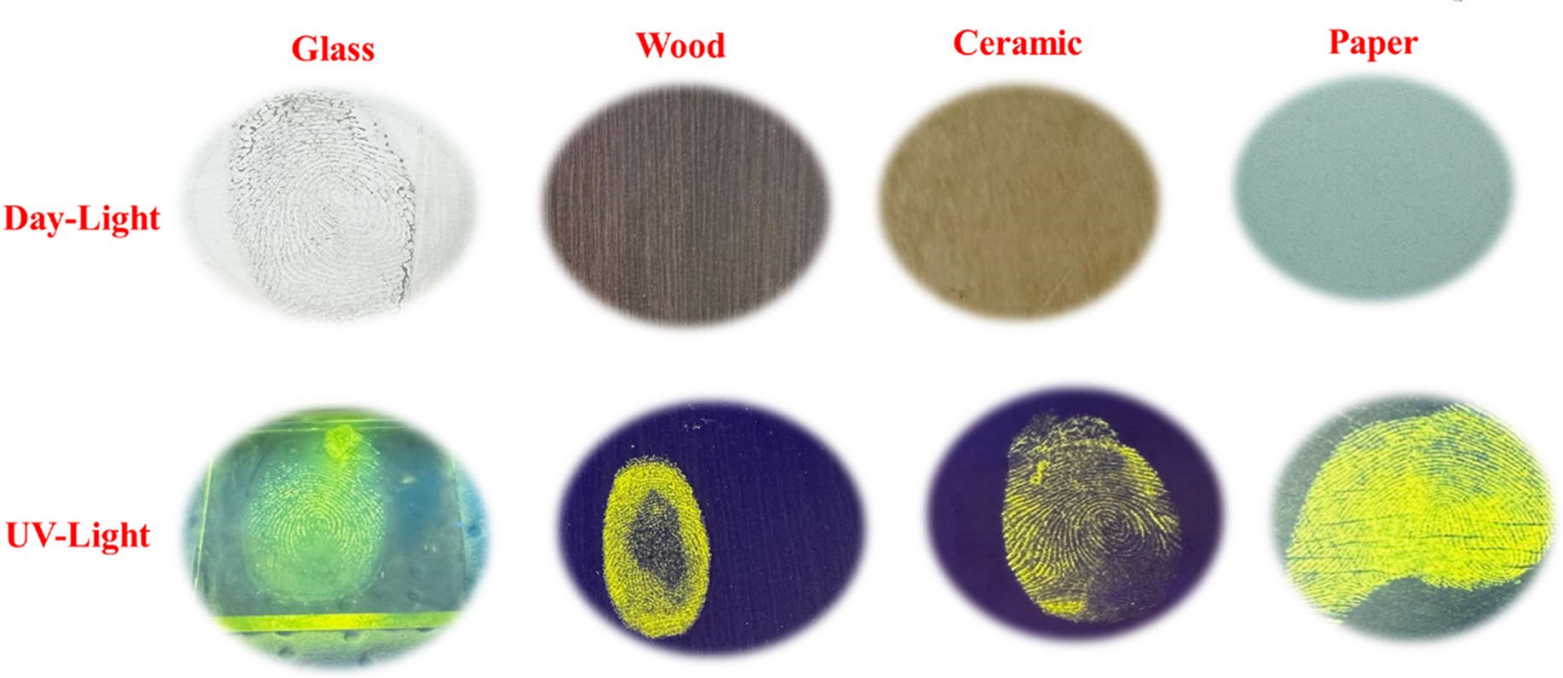
Images of latent fingerprint developed with fluorescent **MR-1** on porous and non-porous surfaces

Figure 8 displays latent fingerprints developed on glass, wood, ceramic, and paper surfaces using the fluorescent dye MR-1. Under visible light, fingerprint patterns were faint or undetectable due to poor contrast with the background, particularly on light-colored or porous materials [35–38]. When illuminated with UV light at 365 nm, however, the **MR-1** dye produced a strong yellow-green fluorescence that clearly revealed ridge details across all tested surfaces. The enhancement was most pronounced on non-porous substrates such as glass and ceramic, where fingerprint residues remained at the surface, allowing effective interaction with the dye. On porous materials like wood and paper, reduced clarity was observed, likely due to partial absorption of the residue into the substrate. These findings highlight the potential of **MR-1** for non-destructive, high-contrast fingerprint visualization, especially on smooth, non-porous surfaces commonly encountered in forensic investigations.

### Digital Magnifications of Developed Fingerprint for MR-1

**Fig. 9:**
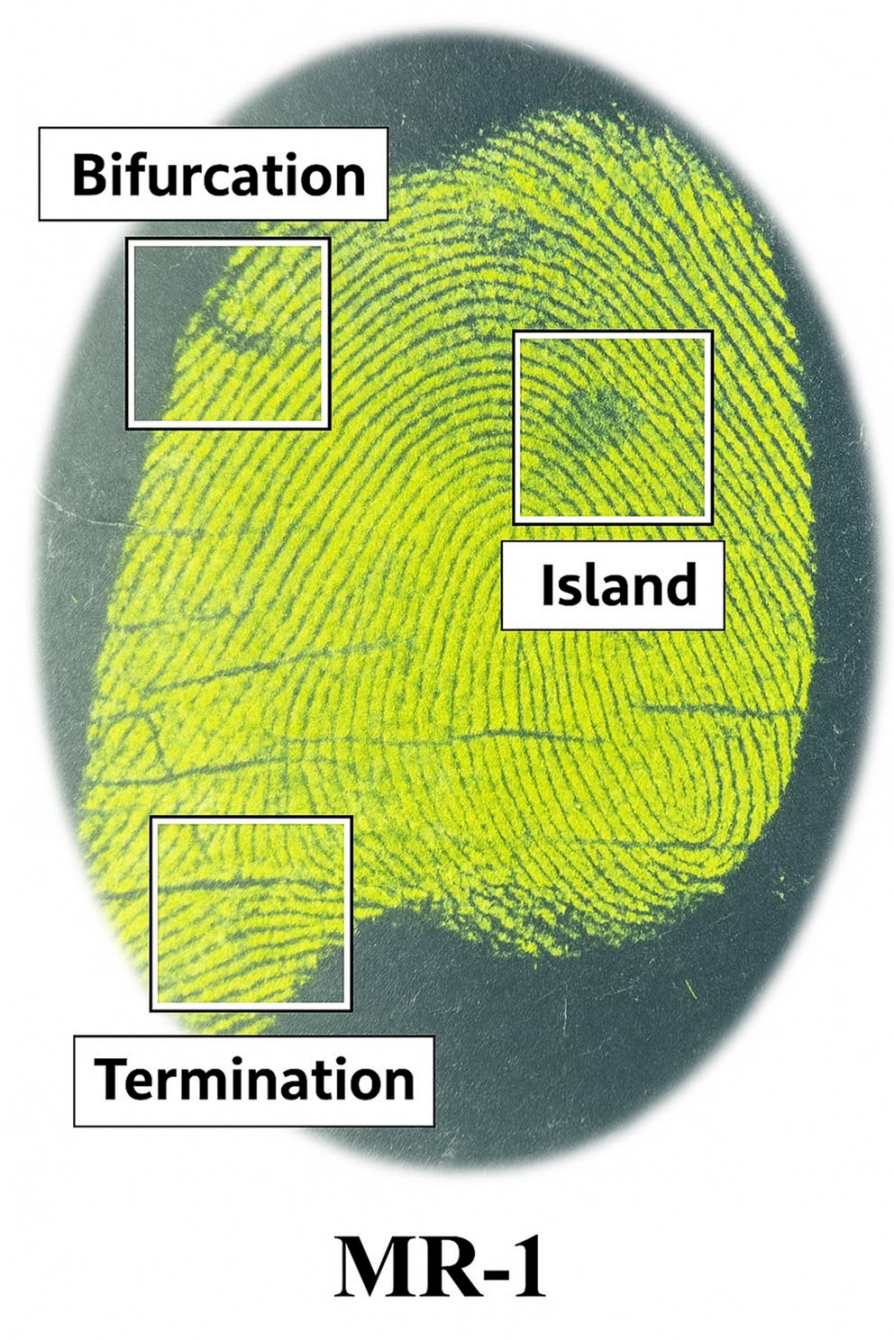
Images of latent fingerprint developed on stainless steel (metallic surface)

Recognition of personal identity relies heavily on the accurate comparison of latent fingerprints collected from crime scenes with reference prints of individuals. This process depends on the clear visualization of friction ridge details, commonly referred to as minutiae, which include distinctive features such as bifurcations, ridge endings (terminations), and islands. In this study, fingerprints were developed on a stainless-steel surface using fluorescent microparticles based on the **MR-1** dye and visualized under UV illumination at 365 nm as shown in Fig. 9. The high fluorescence intensity of **MR-1** enabled the capture of well-defined ridge patterns with excellent contrast against the background. As shown in Fig. 9, digital magnification of the developed fingerprint reveals clear structural minutiae essential for identification. Specific features such as bifurcation, island, and termination are distinctly visible within the highlighted regions, confirming the suitability of **MR-1** for forensic fingerprint imaging. The ability to resolve these fine details under UV light highlights the potential of **MR-1-**based powders in non-destructive and high-accuracy biometric applications.

### Fluorescent Security Ink Performance of MR-2 Under Ambient and UV Light

**Fig. 10:**
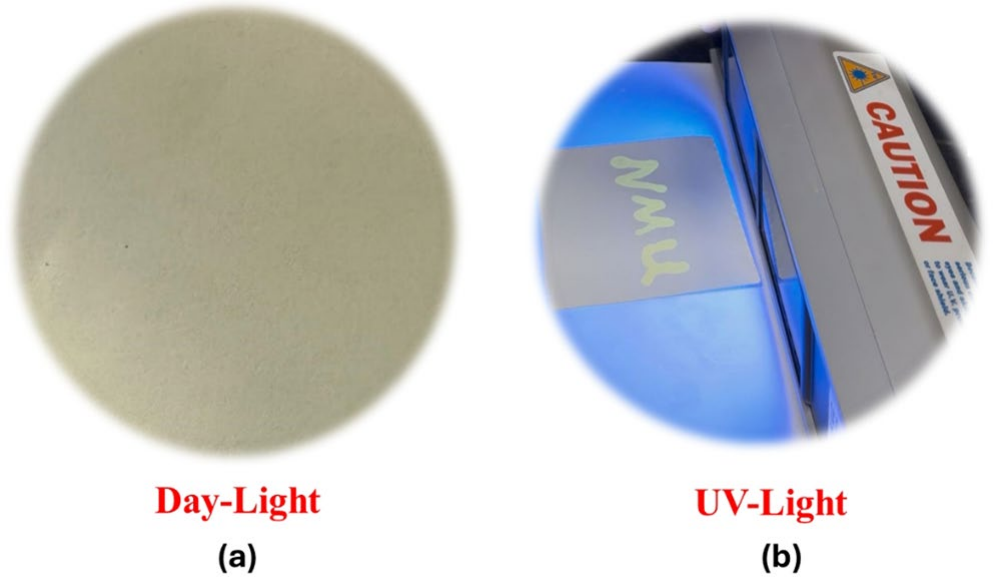
Information loaded on security paper using the ink formulation: **a**) invisible under daylight and **b**) bluish-green fluorescence under 365 nm UV light

**MR-2**-based ink showed strong and stable fluorescence, making it a promising candidate for secure printing. To test its practical use, handwritten text was applied to UV-dull security paper using the ink three months after its preparation. As shown in Fig. 10, the writing remains invisible under normal lighting conditions but becomes clearly visible under UV light at 365 nm, emitting a bright bluish-green glow. This strong contrast demonstrates the ink’s ability to conceal information until deliberately revealed. Notably, the fluorescent signal remained unchanged after more than 30 days of storage at room temperature, indicating excellent long-term stability. These results suggest that **MR-2** ink could be effectively used in applications such as anti-counterfeiting, document authentication, and secure data labeling, where reliable and hidden information is required.

### Conclusion

This work presents the design and synthesis of oxadiazole-based fluorescent dyes with tunable photophysical properties and practical relevance in forensic and security applications. The dyes exhibit strong emission across a wide spectral range, large Stokes shifts, and pronounced solvatochromism, governed by intramolecular charge transfer. These behaviors were supported by density functional theory calculations, which revealed distinct frontier orbital distributions consistent with donor–acceptor architectures. Thermal analysis confirmed the stability of the materials under operational conditions. When incorporated into oxadiazole-based microparticles, the dyes enabled high-contrast visualization of latent fingerprints on a range of surfaces. In parallel, ink formulations demonstrated clear, stable fluorescence under UV illumination while remaining invisible under ambient light highlighting their potential as covert security markers. Together, these findings demonstrate how molecular design strategies can be leveraged to achieve multifunctional fluorescent materials, bridging fundamental photophysics with real-world applications in anti-counterfeiting and forensic science.

## Electronic Supplementary Material

Below is the link to the electronic supplementary material.

Supplementary Material 1

## Data Availability

Data is provided within the manuscript or supplementary information files.

## Declarations

### Dual Publication

The results/data/figures in this manuscript have not been published elsewhere, nor are they under consideration (from you or one of your Contributing Authors) by another publisher.

### Third Party Material

All of the material is owned by the authors and/or no permissions are required.
