# Supplementary material for: Oxadiazole-Based Fluorescent Dyes: Photophysical Characterization and Dual Application in Latent Fingerprint Detection and Security Inks
Source: J Fluoresc. 2025 Jul 23;35(12):12361–72. doi: 10.1007/s10895-025-04441-5 (PMC12858573; doi:10.1007/s10895-025-04441-5)
Supplement: Supplementary file 1 — Supplementary Material 1 [file 10895_2025_4441_MOESM1_ESM.docx]

**Supplementary file**

1. **Materials**

Alfa Assar and Sigma Aldrich supplied the starting materials. The melting points were measured in degrees Celsius using a Gallenkamp electric melting point instrument and are uncorrected. Thermo Scientific Nicolet iS10 FTIR spectrometer was used to get IR spectra (KBr), while a Burker NMR spectrometer was used to obtain NMR spectra in DMSO-*d6* at frequencies of 400 MHz (^1^H-NMR) and 100 MHz (^13^C- NMR). A high-performance twin beam spectrophotometer (T80 series) was used to acquire UV–visible spectra, while a Perkin Elmer 2400 analyzer was used for elemental analysis.

*2.Theoretical calculations*

Theoretical calculations were performed with Density Functional Theory (DFT) using the Gaussian 09 Package. The final geometry optimization and energy level of the molecular orbital HOMO (Highest Occupied Molecular Orbital) and LUMO (Lowest Unoccupied Molecular Orbital) were calculated using the B3LYP/6-31G (d) function and basis set. The electron density and spatial orientation of the HOMO and LUMO molecular orbitals and electrostatic potential (MEP) maps were also calculated by the same function. Electronic transitions were calculated using ZINDO and STO-6G basis set in ArgusLab software from optimized geometry by B3LYP/6-31G (d) in Gaussian 09. Theoretical calculations were all performed in water and gas phase

1. *Thermogravimetric analysis*

The thermogravimetric analysis (TGA) was performed using a thermogravimetric analyzer equipment (Perkin-Elmer THA-1), under nitrogen atmosphere with gas flow of 20 mL/min. The sample with initial mass ranged from 5.124 to 6.224 mg was analyzed in a platinum crucible, with temperature ranged from 20 to 600 ◦C and heating rate of 10 ◦C/min.

1. *Latent fingerprint detection*

The visualization of latent fingerprint was conducted using the developed fluorescent microparticles. The fingerprint samples were impressed on different types of surfaces common to forensic cases (paper, wood, ceramic, and glass). A brush (TIGRE®, synthetic fiber) was contacted with fluorescent microparticles and applied carefully over the fingerprint samples. The excess dust on the fingerprints was removed by shaking the object containing fingerprints and using a thermal blower (without heating). The images of processed fingerprints were obtained under ultraviolet light of 365 nm by using a digital camera Samsung® Galaxy J5 Pro. The substrates were stored under environmental conditions.

**2. Analytical Measurements**


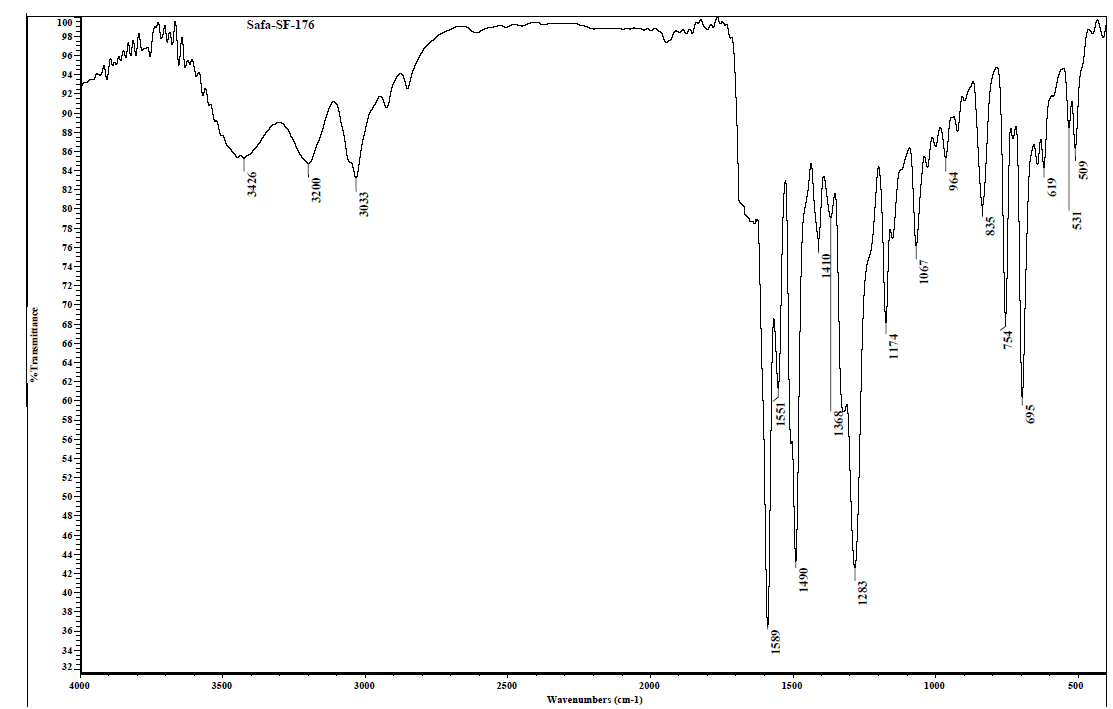


**Figure (S1): IR spectrum of compound MR-1.**


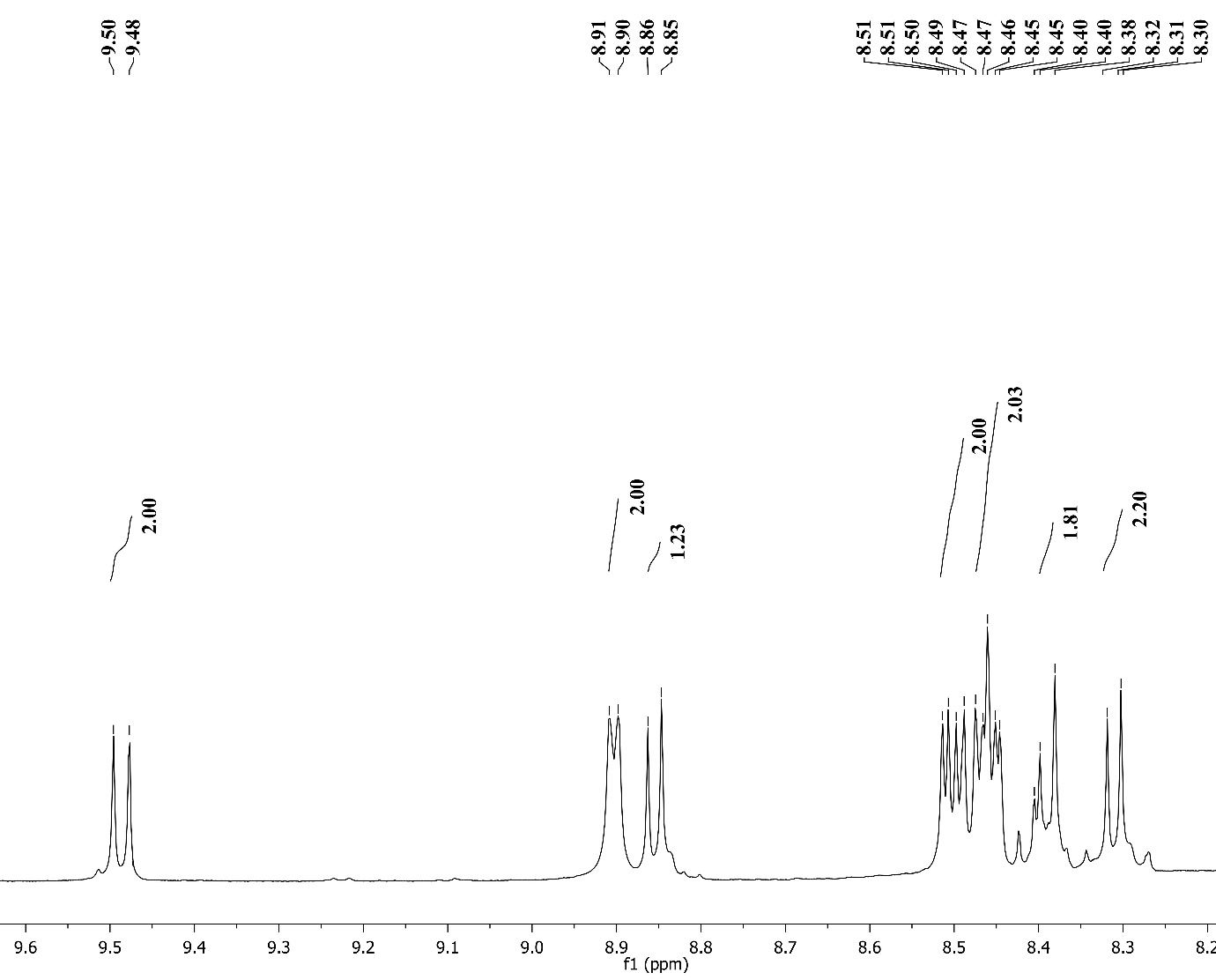


**Figure (S2): ^1^H NMR spectrum of sensitizer MR-1.**


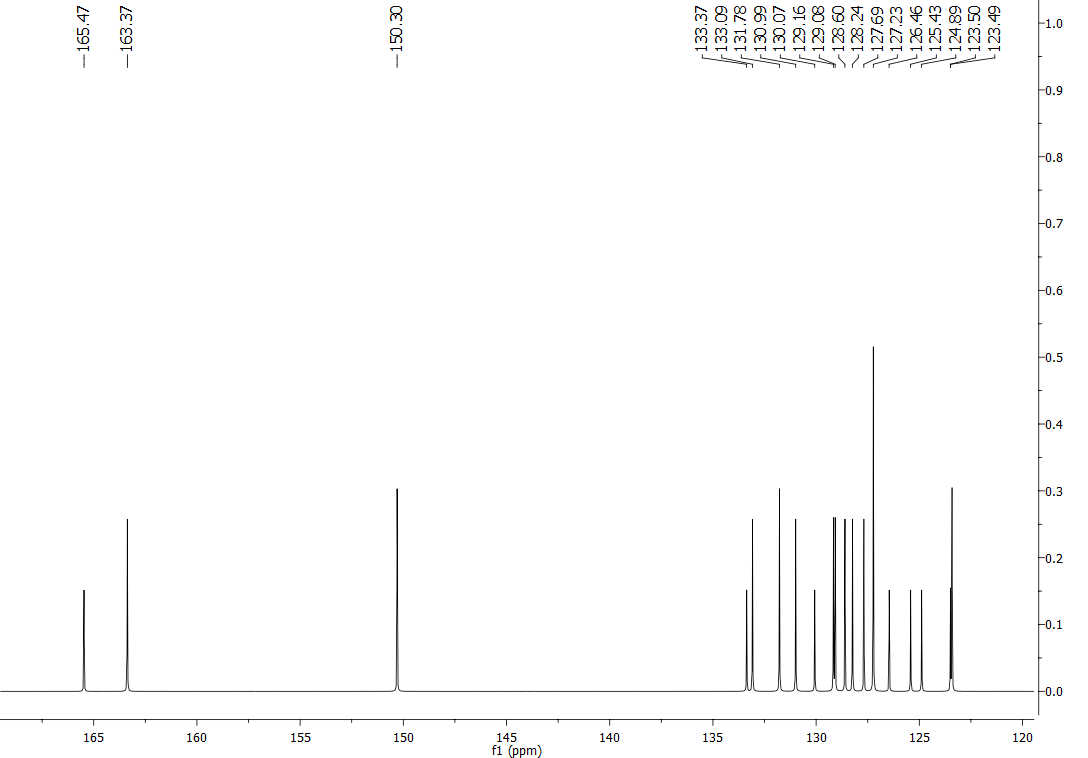


**Figure (S3): C^13^ NMR spectrum of sensitizer MR-1.**


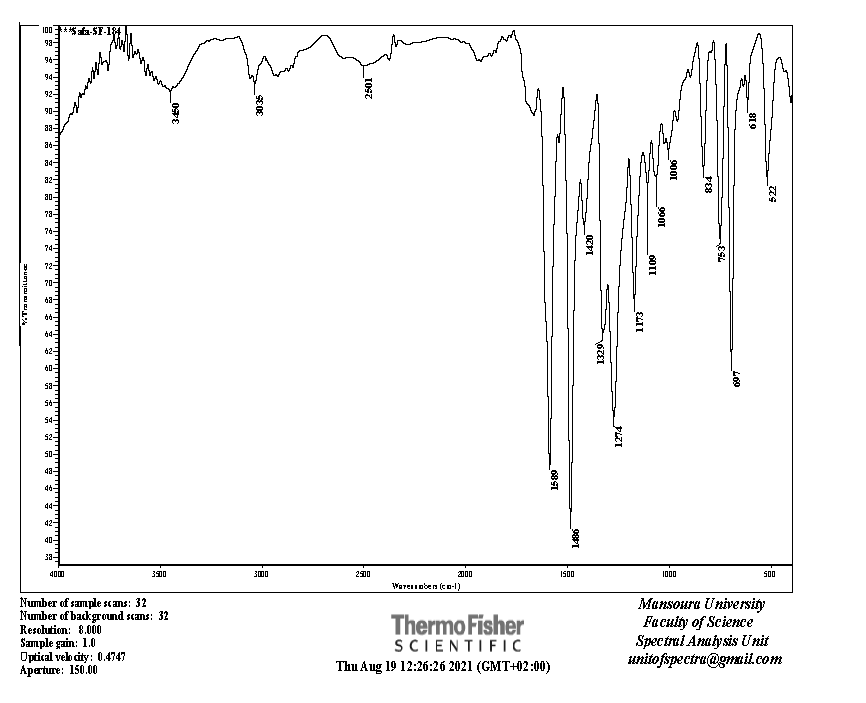


**Figure (S4): IR spectrum of compound MR-2.**


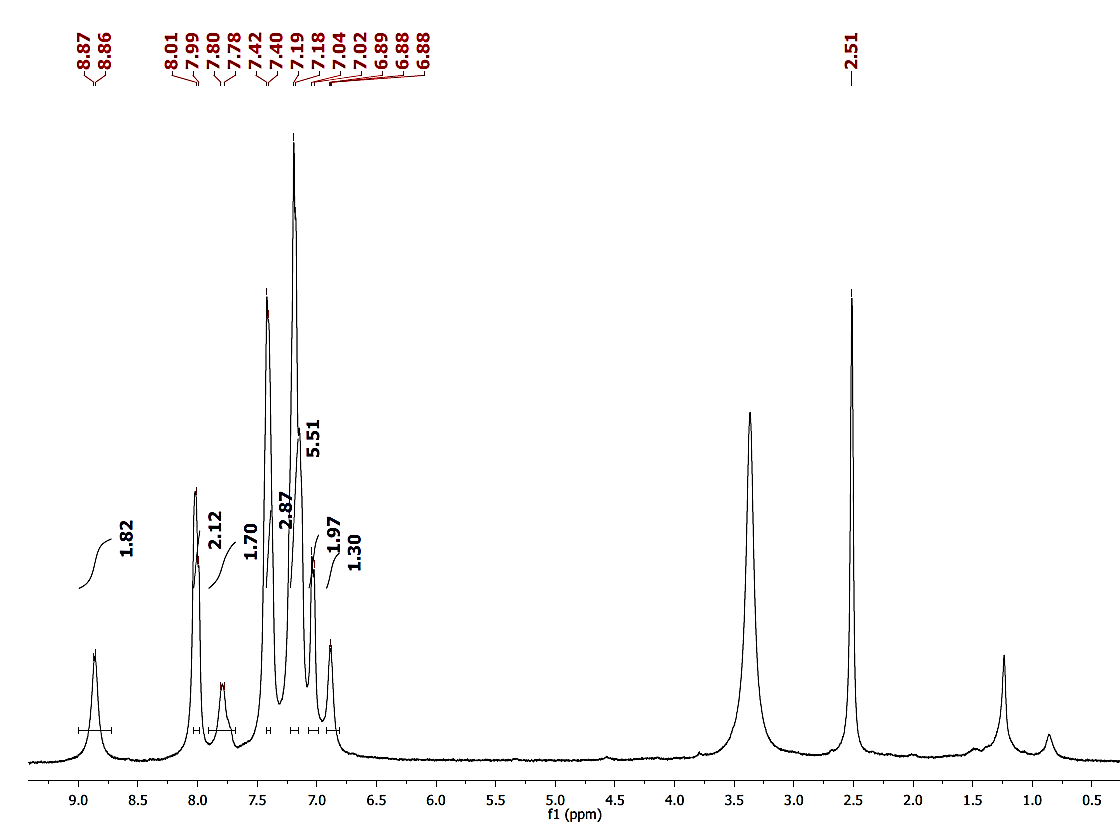


**Figure (S5): ^1^H NMR spectrum of sensitizer MR-2.**


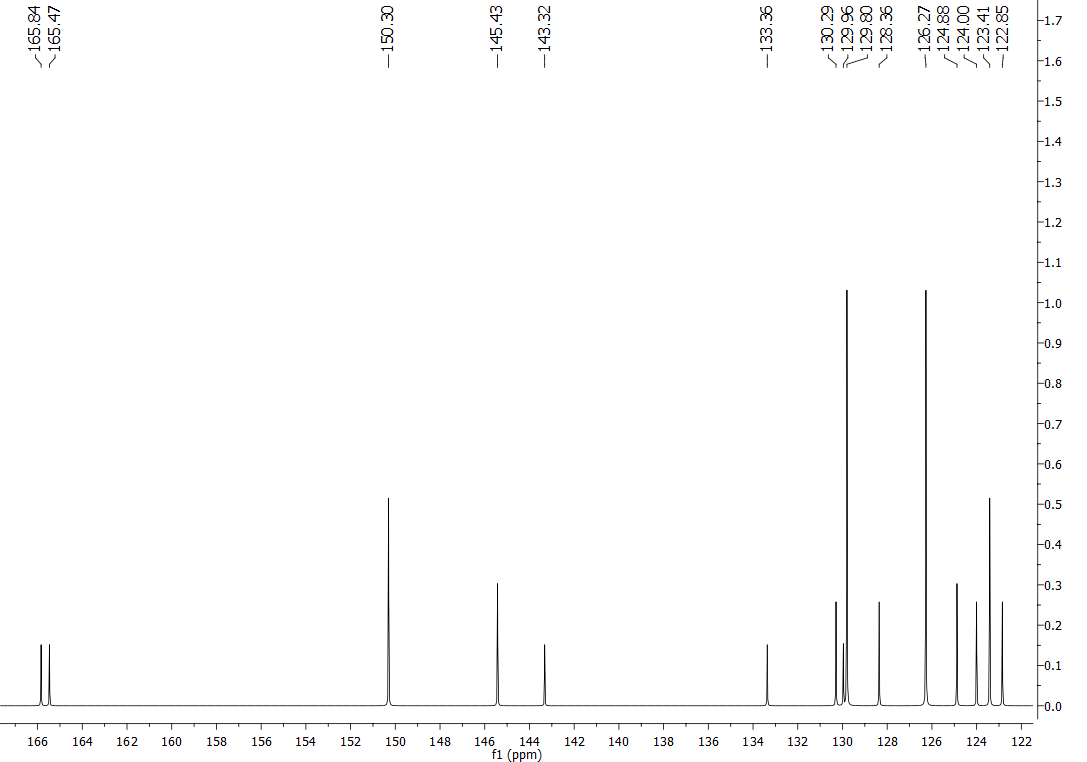


**Figure (S6): C^13^ NMR spectrum of sensitizer MR-2.**
